# Neutrophil-Fibroblast Crosstalk Drives Immunofibrosis in Sequelae of Pelvic Inflammatory Disease Through Neutrophil Extracellular Traps

**DOI:** 10.1155/mi/3113542

**Published:** 2025-11-11

**Authors:** Chunxiao Dang, Jinxing Liu, Xiao Yu, Xian Wang

**Affiliations:** ^1^First Clinical Medical College, Shandong University of Traditional Chinese Medicine, Jinan, Shandong, China; ^2^Department of Gynecology, Affiliated Hospital of Shandong University of Traditional Chinese Medicine, Jinan, Shandong, China

**Keywords:** aseptic inflammation, fibroblast, fibrosis, neutrophil extracellular traps, sequelae of pelvic inflammatory disease

## Abstract

**Background:**

Due to the complex pathogenesis of sequelae of pelvic inflammatory disease (SPID), targeted therapeutic agents are still lacking. Here, we investigated the interactions between neutrophils and uterine fibroblasts (FBs) in developing tissue fibrosis in SPID.

**Methods:**

A rat model of SPID was constructed to assess the roles of autophagy and neutrophil extracellular traps (NETs) in SPID rats. Single-cell sequencing data from the public database GSE223639 were utilized to identify the specific cell cluster FBs where NETs act. A DMSO-induced HL-60-driven neutrophil-like (dHL-60) cell model was established, and neutrophil-like cells were treated with rapamycin and MHY1485 to activate and inhibit autophagy, respectively, to observe the differences in the production of NETs. NETs were cocultured with FBs to observe the effects on FB proliferation, migration, apoptosis, and phenotypic transformation.

**Results:**

In vivo experiments revealed that there was a consistency in the expression of autophagy and NETs in the adherent tissues of rats with the SPID model and that autophagy promotes the generation of NETs, which are collectively involved in the fibrosis of pelvic tissues in SPID. Single-cell sequencing identified FBs, the cells in which NETs play a major role in aseptic inflammation. Further in vitro studies confirmed that NETs inhibit FB apoptosis while promoting horizontal and longitudinal migration, phenotypic transformation, and hyperproliferation of FBs, thereby exacerbating tissue fibrosis.

**Conclusions:**

Autophagy promotes the generation of NETs, which facilitates FB transformation and hyperproliferation and exacerbates the degree of adhesion and fibrosis in the pelvic tissue of SPID.

## 1. Introduction

Sequelae of pelvic inflammatory disease (SPID) is a series of sequelae such as destruction of the female upper genital tract and its surrounding tissues, extensive adhesions, hyperplasia, and scar formation, etc., occurring as a result of pelvic inflammatory disease that has not been correctly treated in a timely manner [1]. It is characterized by a long course, intractability, and recurrent episodes, manifested by chronic pelvic pain, infertility, and ectopic pregnancy, which seriously affects women's reproductive health and aggravates direct and indirect medical costs [1, 2].

Modern studies have shown that the presence of pathogenic bacteria is almost absent in the pelvic tissues of patients with SPID, which is mainly characterized by tissue damage, adhesions and hyperplasia secondary to infection by bacteria and other pathogens, and tissue fibrosis is one of the main manifestations of SPID. Neutrophil extracellular traps (NETs) contain chromatin bound to granzymes, which are associated with inflammation and fibrosis [3]. Several studies have demonstrated significant neutrophil activation in peripheral blood or diseased tissues of patients with chronic inflammatory diseases, accompanied by enhanced NETs and impaired NETs degradation [4, 5]. For example, NETs can promote the formation of epidural fibrosis after spinal surgery, tissue fibrosis in patients with systemic lupus erythematosus, and pulmonary fibrosis [6–8]. NETs are induced by a variety of pathogenic factors and proinflammatory stimuli, and autophagy is one of the important regulators of the NETs formation process [9, 10]. However, the mechanisms involved in reducing the generation of NETs and attenuating pelvic tissue fibrosis by inhibiting autophagy are currently unknown.

Single-cell sequencing enables high-resolution analysis of gene expression at the individual cell level, providing insight into cellular heterogeneity, developmental processes, and disease mechanisms [11–13]. Our study initially confirmed that autophagy and NETs are jointly involved in the process of SPID tissue fibrosis by constructing a SPID rat model. Second, by applying single-cell sequencing, we found that NETs in aseptic inflammation mainly interacted with fibroblasts (FBs). Subsequent in vitro studies showed that autophagy promoted the release of NETs from neutrophils, and excessive NETs led to phenotypic transformation and overproliferation of FBs, which aggravated the hyperplasia and adhesion of pelvic tissues in SPID model rats.

## 2. Materials and Methods

### 2.1. Rats and Treatment

Body mass (220 ± 20) g of SPF-grade female Wistar rats, 12 weeks old, were purchased from Shandong Ponyue Animal Laboratory Company. The rats were housed at (22 ± 2)°C, (50% ± 20%) humidity, and 12/12 h light/dark cycle. The Ethics Committee for Animal Experiments of Shandong University of Traditional Chinese Medicine approved the experiment (reference number SDUTCM20241114223).

According to a previous study [14], the SPID rat model was constructed using exercise fatigue, starvation-assisted *E. coli* upstream infection method, and the Control group was fed normally. After successful modeling, the model rats were randomly divided into the Model group, the MHY1485 group (10 mg/kg MHY1485 suspension), and the RAPA group (2 mg/kg rapamycin suspension), and the suspension was injected intraperitoneally every other day for a total of 14 days. During this period, they were fed normally.

### 2.2. Histological Staining

After anesthetizing the rats, the fallopian tubes on both sides were removed, and the surrounding fat, tissue, and surface membrane were separated. The uterus, fallopian tubes, ovaries, and pelvic connective tissue adhesion sites were retained. After rinsing with PBS, the adhesion tissue was fixed with 4% paraformaldehyde, followed by a dehydration process. Next, the adherent tissues were embedded in paraffin and sectioned using a slicer. Sections were mounted on slides and subjected to dewaxing and rehydration. Subsequently, hematoxylin and eosin (H&E) staining and Masson staining were performed. Finally, the stained sections were scanned using an optical scanner.

### 2.3. Single-Cell RNA Sequencing Analysis

We obtained scRNA-seq data from the GSE223639 dataset of the GEO database, including seven samples and seven normal tissue samples (https://www.ncbi.nlm.nih.gov/geo/query/acc.cgi?acc=gse223639) [15]. Data were preprocessed using the Seurat software package, selecting only genes expressed in at least three cells and one cell expressing at least 200 genes and retaining cells with <15% of mitochondrial genes and gene counts between 500 and 6000 [16].

After quality control and normalization of the data, the ‘FindVariableFeatures' function was used to identify the top 2000 highly variable genes [17, 18]. Subsequently, principal component (PC) analysis (PCA) was performed to determine the optimal number of PCs. JackStraw analysis was used to identify significant PCs and appropriate PCs were selected for cell clustering analysis based on the variance ratio. To improve annotation accuracy, FindAllMarkers was used to annotate and visualize major cell types based on reference datasets from the CellMarker database and published literature [19]. Finally, we examined hub gene expression in all cell clusters and explored cellular crosstalk using the ‘CellChat' software package [20].

### 2.4. Cell Culture

Human promyeloid leukemia cell HL-60 (RRID: CVCL_0002) (BC-C-HU-064, Bio-Channel, China) was cultured in Iscove-modified Dulbecco medium (IMDM) (2440053, Gibco, USA) containing 20% heat-inactivated fetal bovine serum (FBS) (S711-001S, Lonsa Science SR, Uruguay). As in previous studies [21–23], to prepare DMSO-induced HL-60-driven (dHL-60) cells with a neutrophil phenotype, we exposed HL-60 cells to 1.25% DMSO at a density of 1 × 10^6^ cells/mL for 5 days (37°C, 5% CO_2_). All cell lines underwent rigorous quality control prior to experimental use. Identity was authenticated through short tandem repeat (STR) profiling (performed according to International Cell Line Authentication Committee [ICLAC] standards) and cross-referenced with the Cellosaurus database. Meanwhile, species-specific PCR confirmed the absence of interspecies cross-contamination. The cells are completely devoid of mycoplasma and bacterial contamination.

### 2.5. Induction and Extraction of NETs

Adjust the density of dHL-60 cells to 1 × 10^6^ cells per well using serum-free IMDM medium, and seed them into a 6-well plate in four groups (Control group, PMA group, MHY1485 + PMA group, and RAPA + PMA group). The Control group was not treated with PMA. The PMA group was stimulated with 100 nM PMA (IP1010, Solarbio, China) for 4 h. During the induction of NETs, to induce or inhibit autophagy, dHL-60 cells were treated with 10 nM autophagy inducer (rapamycin; AY-22989, MCE, USA) and 10 μM autophagy inhibitor (MHY1485; HY-B0795, MCE, USA) for 4 h. After stimulation, the medium was removed, and the wells were washed with IMDM. To collect the NETs adhering to the bottom, 1 mL of PBS was added to each well to wash it carefully twice and resuspended in IMDM. To minimize contamination from cell debris while preserving NET activity, the suspension was centrifuged at 450 × *g* for 10 min at 4°C to remove whole cells and fragments [24]. The NET-enriched supernatant was then collected. The collected supernatant was either used immediately or stored at −80°C until use.

### 2.6. NETs-FB Coculture

Rat uterine FBs (FH-R128, FuHeng BioLogy, China) were resuspended in complete medium (CM-R054, Procell, China) and seeded into 6-well plates at a density of 1 × 10^6^ cells per well. The cells were then treated with the four different NETs supernatants collected in Section 2.5 and incubated for 24 h at 37°C.

### 2.7. Scanning Electron Microscopy

The PMA-treated dHL-60 cells were fixed with 2.5% glutaraldehyde for 24 h at 4°C. After standard dehydration and sputter coating processes, the generation of NETs by each group of cells was observed under a scanning electron microscope (SU8100, HITACHI) at an accelerating voltage of 2.0 kV.

### 2.8. Immunofluorescence

Paraffin sections or cell crawls were subjected to antigen repair and closed with 5% bovine serum albumin (BSA) (GC305010-50 g, Servicebio, China). Subsequently, they were incubated with primary antibodies (CitH3 1:500, MPO 1:100, ATG5 1:100, LC3B 1:500) overnight at 4°C. Next, incubated with goat antirabbit secondary antibody (ab150077, Abcam) for 1 h at room temperature. Cell nuclei were restained using DAPI (C0065, Solarbio, China). Sections were observed and captured using a fluorescence microscope.

### 2.9. Quantitative Real-Time Polymerase Chain Reaction (qRT-PCR)

Total RNA was extracted from FBs using the Cellular RNA Rapid Extraction kit (400-100, Goonie, China) according to the manufacturer's instructions. To quantify Col Ⅰ, Col Ⅲ, MMP-9, and α-SMA expression, total RNA was reverse transcribed to cDNA using the Rapid Reverse Transcription kit (500-101, Goonie, China), followed by qRT-PCR using a qPCR kit (500-101, Goonie, China). The relative expression of Col Ⅰ, Col Ⅲ, MMP-9, and α-SMA was expressed as a function of threshold cycling (Ct) and analyzed by the 2^−ΔΔCt^ method. PCR primers used in this study are listed in Supporting Information 1: Table S1.

### 2.10. Western Blotting (WB)

FBs were lysed on ice using RIPA lysis buffer (R0010, Solarbio, China). Protein concentration was determined using BCA protein assay kit (PC0020, Solarbio, China). Proteins were separated by SDS-PAGE and transferred to polyvinylidene difluoride (PVDF) membranes (IPVH00010, Merck, Germany). The membranes were then incubated with primary and secondary antibodies. Protein blot images were captured using a chemical illumination image analysis system (Tanon 5200, Tanon, China). The intensity of the bands was quantified using Image J software. Details of the antibodies used in this study are listed in Supporting Information 2: Table S2.

### 2.11. Cell Counting Kit 8 (CCK8) Test

FBs from each well of the six-well plate were inoculated into the 96-well plates with a density of 3000 cells per well and cultured in complete medium. Cell viability was detected at 0, 24, 48, and 72 h using CCK8 solution (E-CK-A362, Elabscience, China) according to the manufacturer's protocol description.

### 2.12. Transwell Invasion Test

Transwell chambers (356234, Corning, USA) precoated with Matrigel matrix were used to detect cell invasion capacity. FBs were inoculated into the upper chamber at a density of 1 × 10^5^ cells/well in 200 µL serum-free medium, and 500 μL corresponding medium containing 20% FBS was added to the lower chamber to induce FBs to move down. FBs were incubated in the cell incubator for 24 h, and the nonmigrating and noninvasive cells in the upper chamber were gently wiped with a cotton swab. Then FBs were fixed with 4% paraformaldehyde for 10 min and stained with 0.1% crystal violet (G1062, Solarbio, China). Three random fields of view of Transwell chambers were collected using an inverted microscope (AXIO Vert. A1, Zeiss, China), and the number of cells in the lower chamber was calculated.

### 2.13. Flow Cytometry Analysis

Apoptosis analysis was performed using the Annexin V-FITC/7-AAD Apoptosis Detection kit (100-105-30, Goonie, China). Treated FBs were collected and centrifuged at 2000 rpm for 5 min. About 5 × 10^4^ cells were taken and resuspended in binding buffer (500 μL). Then, the cells were incubated with Annexin V-FITC (5 μL) and 7-AAD (5 μL) for 20 min at room temperature away from light. The apoptosis of FBs was analyzed using flow cytometry.

### 2.14. Statistical Analysis

Data from two independent experiments were expressed as standard deviation (SD) ± mean and analyzed by SPSS.26. Due to the small sample size, formal normality tests were not conducted [25, 26]. To ensure reliable test validity, one-way ANOVA was first performed for multigroup comparisons. If overall differences existed (*p* < 0.05), Fisher's least significant difference (LSD) post-hoc test was further applied for planned pairwise comparisons, with *p*  < 0.05 indicating statistically significant differences. Fisher's LSD method was chosen to maintain sufficient statistical power while controlling type I errors, which is particularly crucial for our exploratory study with limited sample size.

## 3. Results

### 3.1. Evaluate the Effects of Autophagy and NETs in Model Rats

**Figure 1:**
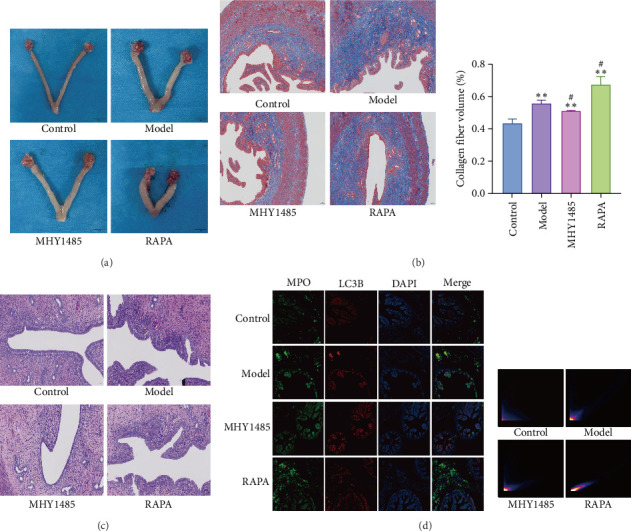
Degree of tissue fibrosis in rats with autophagy and NETs-exacerbated SPID model (*n* = 3). (A) Changes in the gross morphology of the uterus, fallopian tubes, and ovaries of rats. (B) Masson staining and collagen fiber volume ratio of rat oviduct adhesion tissue (10×). (C) H&E staining of rat oviduct adhesion tissue (20×). (D) Immunofluorescence staining and colocalization of MPO and LC3B in rat oviductal adherent tissues (×20). The Pearson correlation coefficient (PCC) was 0.03 in the Control group; PCC was 0.59 in the Model group; PCC was 0.62 in the MHY1485 group; and PCC was 0.76 in the RAPA group. 0 ≤ PCC < +0.5 indicates no significant colocalization relationship, while + 0.5 < PCC ≤ +1 indicates a positive correlation between the two fluorescent signals. *⁣*^*∗∗*^*p* < 0.01 versus Control; *⁣*^#^*p* < 0.05 versus Model.

The role of autophagy and NETs in SPID rats was assessed by H&E staining, Masson staining, and immunofluorescence, as shown in Figure 1. Compared with the Control group, the Model group showed thickening and edema of the fallopian tubes, thickening and hardening of the uterine end of the fallopian tubes, and adhesion of the fallopian tubes to the ovaries in clusters or accompanied by cysts. RAPA exacerbated tissue damage, while MHY alleviated tissue damage (Figure 1A). Masson staining caused a blue coloration of the collagen fibers, and the volume ratio of collagen fibers in the Model group was significantly increased compared with that of the Control group, which was exacerbated by RAPA, while MHY attenuated the volume of collagen fibers in the model large (Figure 1B). H&E staining showed that in the Control group, the tubal structure was clear, the lumen was smooth, the cilia were abundant, and no obvious inflammatory cell infiltration was observed. In the Model group, the tubal structure was unclear, the wall was thickened, the cilia were messy and coarsely adhered, and the inflammatory cell infiltration was more obvious. RAPA aggravated this situation, and MHY attenuated the inflammatory infiltration in the Model group (Figure 1C). Immunofluorescence showed that the mean fluorescence intensity of MPO and LC3B was significantly increased in the Model group compared with the Control group, which was exacerbated by RAPA and attenuated by MHY in model rats (Figure 1D). These results suggest that autophagy promotes the generation of NETs and exacerbates the adhesion and fibrosis of the pelvic tissue in SPID.

### 3.2. Single-Cell Sequencing Analysis

**Figure 2:**
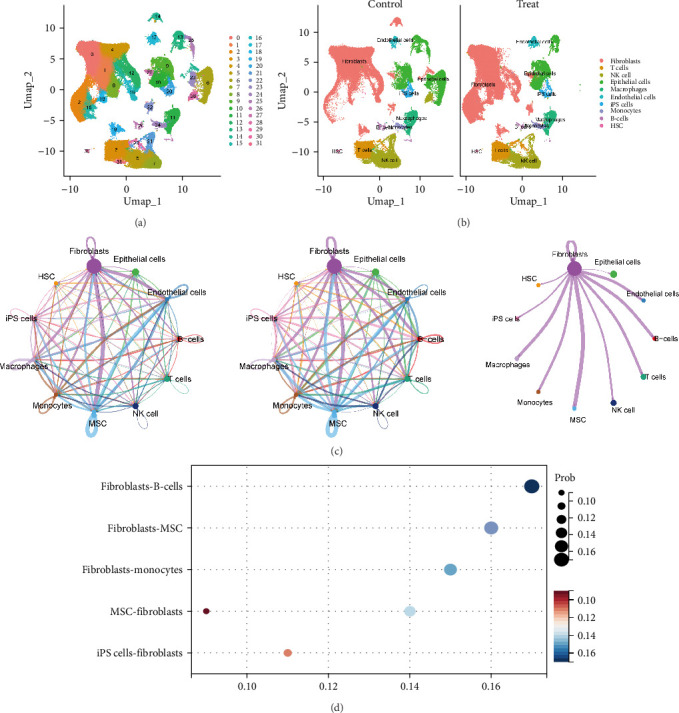
The single-cell transcriptomic atlas of aseptic inflammation in the GSE223639 dataset. (A) UMAP plot showing the 31 cell clusters from the GSE223639 dataset. (B) UMAP plot showing the 10 cell types of the scRNA-seq dataset from aseptic inflammation after annotation. (C) The number and strength of interactions between cell populations. (D) Bubble plot of intercellular communication.

Specific cell clusters where NETs may act were further identified by single-cell sequencing. After data normalization, downscaling, and cell clustering analysis, 154,295 cells were clustered into 31 cell clusters (Figure 2A). Based on the expression of classical marker genes in each cell cluster, 10 cell types were classified: FBs (LUM), T cells (CD3D), NK cells (NKG7), epithelial cells (KRT18), macrophages (AIF1), endothelial cells (EGFL7), iPS cells (SOX17), monocytes (S100A9), B-cells (IGKC), and HSC (TPSAB1) (Figure 2B). In addition, quantitative analysis of each cell type in the samples showed that FBs were overrepresented in the samples. Analysis of cellular communication showed that there were varying degrees of connectivity between all cells, with FBs showing a relatively high number of connections and signal intensity with the other groups (Figure 2C). The top three cellular communications with the highest probability values of FBs as the primary sender were mainly in the MK and MIF pathways, with B-cells and MSCs acting as the receivers. The top three cellular communications with the largest probability values of FBs as primary receivers were mainly in the MK, PTN pathway, with MSC, and iPS cells as senders (Figure 2D, Supporting Information 3: Table S3). Differentially expressed genes between control and treated FBs include genes related to the collagen family (Col12A1, Col8A1, Col24A1, etc.) and matrix metalloproteinases (MMP1, MMP2, MMP3, etc.) (Supporting Information 4: Table S4).

### 3.3. Results of NETs Induction

**Figure 3:**
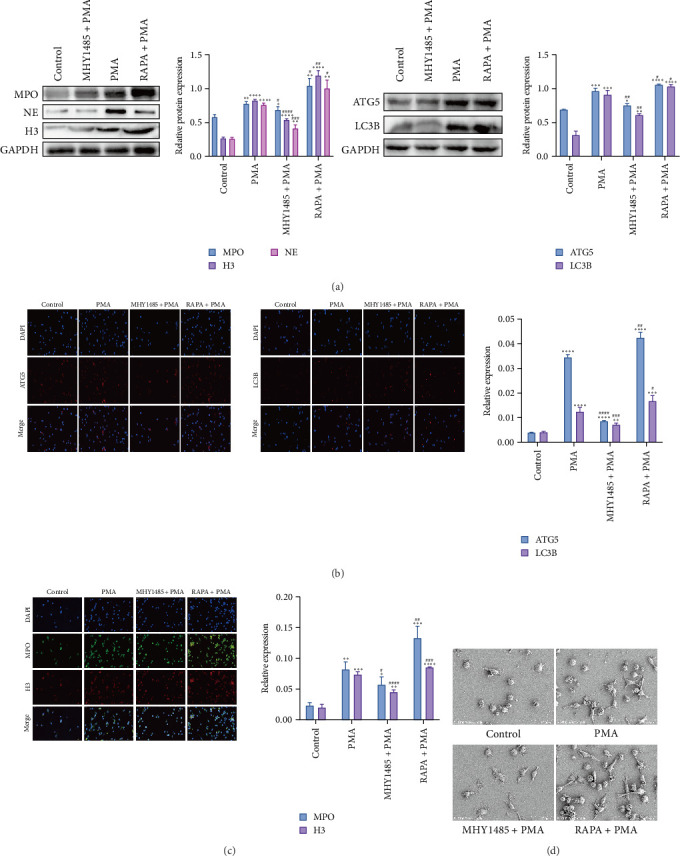
Autophagy promotes the release of NETs from dHL-60 cells. (A) Expression of related proteins in dHL-60 cells (*n* = 3). (B, C) Expression of autophagy markers and NETs markers detected by immunofluorescence (*n* = 3). (D) Scanning electron micrographs of NETs in dHL-60 cells (scale bar: 50 μm). *⁣*^*∗*^*p* < 0.05, *⁣*^*∗∗*^*p* < 0.01, *⁣*^*∗∗∗*^*p* < 0.001 and *⁣*^*∗∗∗∗*^*p* < 0.0001 versus Control; *⁣*^#^*p* < 0.05, *⁣*^##^*p* < 0.01, *⁣*^###^*p* < 0.001 and *⁣*^#####^*p* < 0.0001 versus PMA.

To elucidate the role of autophagy in the release of NETs and the effect of NETs on the proliferation and differentiation of FBs, HL60 cells were treated with 1.25% DMSO and allowed to differentiate into dHL-60 cells for 5 days and then treated with PMA for 4 h. The expression of autophagy-related proteins ATG5 and LC3B as well as NETs-related proteins NE, H3, and MPO was confirmed to be elevated in PMA-treated dHL-60 cells by WB analysis, and the elevation was more obvious in the RAPA + PMA group than that in the PMA group, and the protein expression was decreased by the treatment of MHY1485 (Figure 3A). Immunofluorescence assay was used to detect autophagy level and NETs level, and NETs markers H3 and MPO were labeled in red and green, respectively, and Figure 3B,C showed that the fluorescence expression levels of H3 and MPO were elevated in dHL-60 cells of the PMA group compared with the Control group. Compared with the PMA group, the fluorescence expression level was elevated in the RAPA + PMA group and decreased in the MHY1485+PMA group, and the autophagy markers ATG5 and LC3B were consistent with the expression of NETs markers. Scanning electron microscopy was used to detect the structure of NETs in dHL-60 cells, and the number of NETs that produced a reticular structure after stimulation was increased in dHL-60 cells in the PMA group compared with the Control group. The number of NETs increased after RAPA treatment and decreased after MHY1485 treatment compared with the PMA group (Figure 3D). These results suggest that the generation of NETs is dependent on autophagy.

### 3.4. NETs Promote the Proliferation of FBs

**Figure 4:**
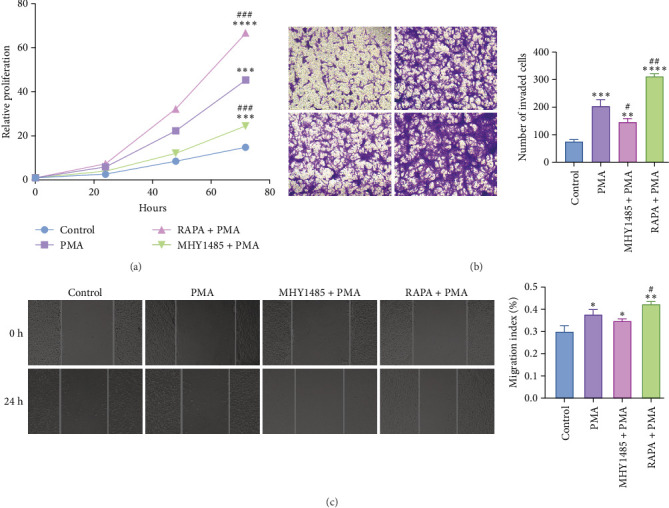
NETs promote fibroblast proliferation and migration. (A) Cell proliferation rate detected by CCK8 assay (*n* = 3). (B) Schematic representation of fibroblast Transwell migration ability (×200) (*n* = 3). (C) Schematic diagram of horizontal migration capacity of fibroblasts (×100) (*n* = 3). *⁣*^*∗*^*p* < 0.05, *⁣*^*∗∗*^*p* < 0.01, *⁣*^*∗∗∗*^*p* < 0.001 and *⁣*^*∗∗∗∗*^*p* < 0.0001 versus Control; *⁣*^#^*p* < 0.05, *⁣*^##^*p* < 0.01, and *⁣*^###^*p* < 0.001 versus PMA.

To demonstrate the role of NETs in tissue fibrosis, we performed CCK8 assay after coculturing NETs with FBs for 0, 24, 48, and 72 h to explore whether NETs could induce FB proliferation in vitro. As shown in Figure 4A, the proliferation rate of FBs was significantly higher in the PMA group at 24, 48, and 72 h compared to the Control group. The cell proliferation rate decreased in the MHY1485+PMA group compared to the PMA group. On the contrary, the cell proliferation rate increased in RAPA + PMA group. It was demonstrated that NETs could promote the proliferation of FBs.

### 3.5. NETs Promoted the Migration of FBs

Scratch assay and Transwell assay were used to detect the effects of NETs on the horizontal and longitudinal migration ability of FBs, respectively. The results of Transwell assay showed that the number of cells crossing the pore membrane was significantly increased in the PMA group compared to the Control group. Compared with the PMA group, the number of cells crossing the pore membrane was significantly reduced in the MHY1485+PMA group, while the number of cells crossing the pore membrane was increased in the RAPA + PMA group (Figure 4B). The results of the scratch experiment showed that the scratch repair rate of 24 h PMA-constituting fiber cells was increased compared with that of the Control group. The scratch repair rate of FBs was increased in the RAPA + PMA group compared with the PMA group, and the scratch repair rate in the MHY1485 + PMA group was not statistically significant (Figure 4C). These results indicated that the migration of FBs was enhanced after NETs treatment compared with the Control group, and NETs could promote the migration of FBs.

### 3.6. NETs Inhibit Apoptosis in FBs

**Figure 5:**
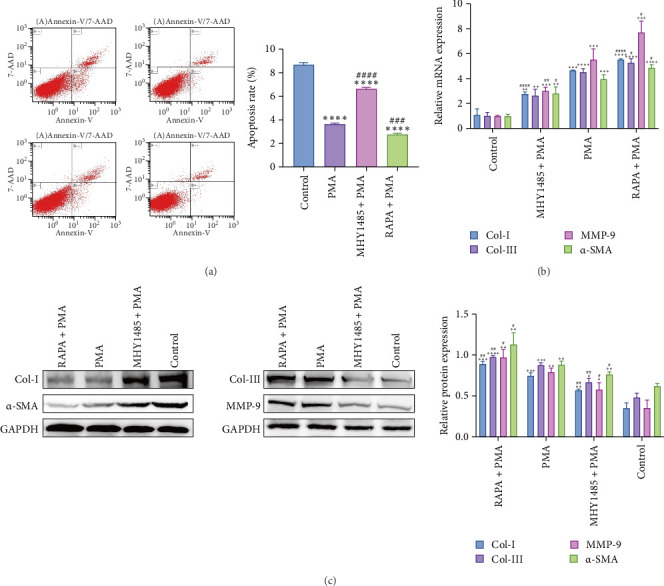
NETs inhibit fibroblast apoptosis and promote fibroblast phenotypic transformation. (A) Schematic diagram of apoptotic state of fibroblasts (*n* = 3). (B) Expression of mRNAs related to phenotypic transformation of fibroblasts (*n* = 3). (C) Expression of fibroblast phenotypic transformation-related proteins (*n* = 3). *⁣*^*∗*^*p* < 0.05, *⁣*^*∗∗*^*p* < 0.01, *⁣*^*∗∗∗*^*p* < 0.001 and *⁣*^*∗∗∗∗*^*p* < 0.0001 versus Control; *⁣*^#^*p* < 0.05, *⁣*^##^*p* < 0.01, *⁣*^###^*p* < 0.001 and *⁣*^####^*p* < 0.0001 versus PMA.

The effect of NETs on apoptosis of FBs was detected by Annexin V-FITC/7-AAD flow cytometry. As shown in Figure 5A, the percentage of apoptotic cells in NETs-treated FBs was reduced compared with the Control group. The percentage of apoptotic cells increased in FBs treated with the MHY1485+PMA group and decreased in FBs treated with the RAPA + PMA group compared with the PMA group. It is suggested that NETs inhibit apoptosis in FBs.

### 3.7. NETs Promoted Phenotypic Transformation of FBs

Finally, we explored the effects of NETs on the expression of proteins and mRNAs related to the phenotypic transformation of FBs. As shown in Figure 5B,C, the protein expression and mRNA expression levels of Col Ⅰ, Col Ⅲ, MMP-9, and α-SMA were upregulated in the NETs group compared with the Control group. Compared with the PMA group, the protein expression and mRNA expression of Col Ⅰ, Col Ⅲ, MMP-9, and α-SMA were downregulated in the MHY1485+PMA group, and the protein expression and mRNA expression of Col Ⅰ, Col Ⅲ, MMP-9, and α-SMA were upregulated in the RAPA+PMA group.

## 4. Discussion

Increasing evidence indicates that neutrophils play a pivotal role in the complex interplay between inflammation and fibrosis [3, 27]. For instance, neutrophils produce tissue inhibitors of metalloproteinases and neutrophil elastase, which collectively activate transforming growth factor-β and recruit other inflammatory cells to the lungs, promoting pulmonary fibrosis [3]. Furthermore, neutrophil-released NETs, through the IL-8 and MMP-9 they carry, jointly activate the profibrotic phenotype of FBs and directly exacerbate extracellular matrix (ECM) degradation, impeding skin wound healing in diabetes [28]. Recent studies indicate that tissue fibrosis development involves complex interactions between immune cells and stromal cells [29], such as activated neutrophils releasing NETs potentially exerting proinflammatory or fibrotic effects by promoting human FB differentiation and activation [30–32]. However, the cellular interactions and functions in SPID remain incompletely understood. Therefore, we employed single-cell sequencing to reveal that in sterile inflammation, NETs primarily target FBs. The differentially expressed genes in FBs include those related to the collagen family (Col12A1, Col8A1, Col24A1, etc.) and matrix metalloproteinases (MMP1, MMP2, MMP3, etc.). Regulatory collagens such as Col12A1, Col8A1, and Col24A1 lead to excessive ECM deposition, forming adhesions [33–35]. Simultaneously, degradative enzymes like MMP1, MMP3, and MMP10, along with their activators, synergize with MMP2 and MMP9 to trigger abnormal ECM degradation and remodeling [36, 37]. This imbalance between synthesis and degradation results in pelvic tissue fibrosis and adhesion formation. However, no experiments have yet validated the specific mechanisms by which neutrophils mediate immunofibrosis in uterine FBs or how these cells interact to cause tissue damage within the inflammatory environment of SPID. Our study reveals that in the SPID model group, pathological changes such as pelvic tissue adhesions and fibrosis worsened, with increased collagen fiber volume. Fluorescent expression of MPO and LC3B in pelvic adhesion tissues showed consistency and significantly higher intensity compared to the Control group. RAPA treatment exacerbated these alterations. This indicates that NETs generated through autophagy promotion aggravate pelvic tissue fibrosis in SPID.

Autophagy is a dynamic process in which autophagosomes fuze with lysosomes and are degraded and cleared by lysosomes in the final stage of the autophagic flux [38]. MHY1485 is an effective cell-permeable mTOR activator that inhibits autophagy by blocking the fusion of autophagosomes with lysosomes [39]. In this study, using a DMSO-induced HL-60 cell model to explore the molecular mechanisms of NET formation, we found that NET formation depends on autophagy, and NET release was reduced after MHY1485 treatment. Additionally, HL-60 cells with fewer passages were more likely to release NETs after PMA induction, consistent with previous studies [9, 10, 40]. NETs are often decorated with a number of protein molecules on their surface, and autophagy is involved in the transport and secretion of some of these proteins [41]. Histone citrullination is an important histone modification process in the formation of NETs, and this process is associated with autophagy [41]. Inhibition of autophagy is detrimental to NETs release and induction of autophagy promotes NETs release.

By coculturing NETs with FBs, we found that NETs increased the proliferative capacity of FBs, promoted horizontal and longitudinal migration, and inhibited apoptosis of FBs while promoting FB-to-myofibroblast transformation. Previous studies have shown that NETs components such as NNA, MPO, NE, and histones release cytokines that lead to inflammation, epithelial mesenchymal transition, epithelial injury, FB activation, and FB myofibroblast transformation, all of which promote the progression of lung fibrosis [42]. Consistent with these findings, our study demonstrated that NETs-treated FBs exhibited significant phenotypic transformation characteristics, as evidenced by the upregulation of Col I, Col III, and α-SMA expression. These proteins are important markers for FB activation, differentiation into myofibroblasts, and participation in ECM remodeling [43, 44]. Myofibroblasts play a key role in wound healing and fibrosis, and they are essential for tissue repair by synthesizing a large number of ECM components and facilitating ECM degradation and remodeling [45]. NETs-induced phenotypic transformation of FBs may accelerate these physiological or pathological processes. Meanwhile, increased expression of MMP-9 suggests that NETs regulate the ability of FBs to degrade ECM [46, 47], which is necessary for tissue remodeling, but overexpression may be associated with tissue destruction and fibrosis progression.

Although our study confirms the role of NETs in regulating FB phenotypic transformation and hyperproliferation, the specific molecular pathways mediating this mechanism remain to be fully elucidated. As previously described, studies in conditions such as inflammatory bowel disease [29, 48] and type 2 diabetes [28] indicate that activated FBs serve as significant sources of chemokines like IL-8, establishing positive feedback loops that recruit and activate neutrophils. Although this study did not directly measure global transcriptional changes or IL-8 levels in FBs, we hypothesize that similar mechanisms likely exist in our SPID model. We hypothesize that NETs may exert direct effects on the ECM via the damage-associated molecular patterns they carry, such as histones and myeloperoxidase, or by activating FB surface receptors, thereby initiating downstream signaling pathways involving molecules like MIF. Elucidating these specific pathways and targets will provide a more detailed molecular mechanism for neutrophil-FB crosstalk and offer novel targets for developing anti-fibrotic therapies.

This study also has some limitations. First, DMSO-induced differentiated HL-60 cells (dHL-60) are a well-established model, but they differ significantly from neutrophils in terms of gene expression and NET responses. Furthermore, while PMA is a reproducible artificial inducer, it bypasses natural stimuli and may not reflect in vivo conditions in SPID. Second, neutrophils are highly susceptible to damage or loss during tissue dissociation and single-cell suspension preparation due to their short lifespan, minimal cytoplasm, low RNA content, and susceptibility to degradation. This leads to their systematic underrepresentation or failure to capture in single-cell RNA sequencing workflows. However, their function is closely linked to macrophages/monocytes. The interactions identified between FBs and monocytes in cellular communication may indirectly reflect the presence of neutrophil-associated inflammatory responses. Finally, the composition and “quality” of NETs may vary depending on the inflammatory environment of each disease. Given that this study employed cell lines for experimental procedures, assessing NET quality in SPID currently presents challenges. Therefore, we encourage future studies using primary rat or human neutrophils, combined with in vivo experiments, to validate findings across a broader range of diseases. In summary, the NET-induced excessive proliferation and migration of FBs, along with the altered expression patterns of the aforementioned proteins, collectively suggest a potential pathological mechanism: activated neutrophils may acquire immunofibrotic effects through the release of NETs, and these fibrotic NETs are able to activate human FBs, inducing them to proliferate, differentiate into myofibroblasts and become immunogenic, a process that is exacerbated by induction of autophagy.

## 5. Conclusion

In the present study, we found that NETs play a bridging role in the communication between neutrophils and FBs during the pathogenesis of SPID, promoting FB overproliferation and phenotypic transformation. The autophagy/neutrophil/FB pathway provides new candidate targets for the design of future diagnostic and therapeutic strategies for SPID.

## Data Availability

The data that supports the findings of this study are available in the Supporting Information of this article.

## Nomenclature

BSA: Bovine serum albumin
CCK8: Cell Counting Kit 8
ECM: Extracellular matrix
NETs: Neutrophil extracellular traps
PCA: Principal component analysis
PCs: Principal components
qRT-PCR: Quantitative real-time polymerase chain reaction
SPID: Sequelae of pelvic inflammatory disease
WB: Western blotting.

## Supporting Information

Supporting Information 1 Table S1: RNA primer sequence.

Supporting Information 2 Table S2: Antibody information.

Supporting Information 3 Table S3: Fibroblasts are the results of the first 3 cell communications between sender and receiver.

Supporting Information 4 Table S4: Partially differentially expressed genes in fibroblasts.
