# Supplementary material for: Neutrophil-Fibroblast Crosstalk Drives Immunofibrosis in Sequelae of Pelvic Inflammatory Disease Through Neutrophil Extracellular Traps
Source: Mediators Inflamm. 2025 Nov 11;2025:3113542. doi: 10.1155/mi/3113542 (PMC12626693; doi:10.1155/mi/3113542)
Supplement: Supporting Information 1 — Table S1: RNA primer sequence. [file 3113542.f1.docx]

| Gene name | Primer name | Primer sequence (5'-3') |
| --- | --- | --- |
| Col-Ⅰ | Col-Ⅰ-F | TGGTCCTGCTGGCAAGAATGG |
|  | Col-Ⅰ-R | TCTGTCACCTTGTTCGCCTGTC |
| Col-Ⅲ | Col-Ⅲ-F | TGGTACTTCTGGTCCTCCTGGTC |
|  | Col-Ⅲ-R | CGCACCGCCTGGCTCAC |
| MMP-9 | MMP-9-F | ACCGCCAACTATGACCAGGATAAG |
|  | MMP-9-R | TGCTTGCCCAGGAAGACGAAG |
| α-SMA | α-SMA -F | CAGGGAGTGATGGTTGGAATGGG |
|  | α-SMA -R | CAGTTGGTGATGATGCCGTGTTC |
| GAPDH | GAPDH-F | CATGACCACAGTCCATGCCA |
|  | GAPDH-R | CAGGGATGATGTTCTGGGCT |

**Table S1.** RNA primer sequence.
