# Supplementary material for: Neutrophil-Fibroblast Crosstalk Drives Immunofibrosis in Sequelae of Pelvic Inflammatory Disease Through Neutrophil Extracellular Traps
Source: Mediators Inflamm. 2025 Nov 11;2025:3113542. doi: 10.1155/mi/3113542 (PMC12626693; doi:10.1155/mi/3113542)
Supplement: Supporting Information 2 — Table S2: Antibody information. [file 3113542.f2.docx]

| **Antibody** | **Manufacturer** | **Article number** | **Dilution ratio** |
| --- | --- | --- | --- |
| ATG5 | ABclonal | A0203 | 1000 |
| LC3B | ABclonal | A19665 | 1500 |
| NE | ABclonal | A13015 | 1000 |
| H3 | ABclonal | A17562 | 1000 |
| MPO | ABclonal | A22900 | 1500 |
| Col-Ⅰ | ABclonal | A1352 | 1000 |
| Col-Ⅲ | ABclonal | A0817 | 1000 |
| MMP-9 | ABclonal | A0289 | 1000 |
| α-SMA | ABclonal | A17910 | 1000 |
| GAPDH | ABclonal | A19056 | 100000 |

**Table S2.** Antibody information.
