# Supplementary material for: Neutrophil-Fibroblast Crosstalk Drives Immunofibrosis in Sequelae of Pelvic Inflammatory Disease Through Neutrophil Extracellular Traps
Source: Mediators Inflamm. 2025 Nov 11;2025:3113542. doi: 10.1155/mi/3113542 (PMC12626693; doi:10.1155/mi/3113542)
Supplement: Supporting Information 3 — Table S3: Fibroblasts are the results of the first 3 cell communications between sender and receiver. [file 3113542.f3.docx]

| **source** | **target** | **ligand** | **receptor** | **prob** | **pval** | **interaction_name** | **interaction_name_2** | **pathway_name** | **annotation** | **evidence** |
| --- | --- | --- | --- | --- | --- | --- | --- | --- | --- | --- |
| Fibroblasts | B-cells | MIF | CD74_CXCR4 | 0.174347582403093 | 0 | MIF_CD74_CXCR4 | MIF - (CD74+CXCR4) | MIF | Secreted Signaling | PMID: 29637711; PMID: 24760155 |
| Fibroblasts | MSC | MDK | NCL | 0.159607025246063 | 0 | MDK_NCL | MDK - NCL | MK | Secreted Signaling | PMID: 28356350 |
| Fibroblasts | Monocytes | MIF | CD74_CD44 | 0.147086777743945 | 0 | MIF_CD74_CD44 | MIF - (CD74+CD44) | MIF | Secreted Signaling | PMID: 29637711; PMID: 26175090 |
| MSC | Fibroblasts | MDK | NCL | 0.138161386799602 | 0 | MDK_NCL | MDK - NCL | MK | Secreted Signaling | PMID: 28356350 |
| iPS cells | Fibroblasts | MDK | NCL | 0.110508992432118 | 0 | MDK_NCL | MDK - NCL | MK | Secreted Signaling | PMID: 28356350 |
| MSC | Fibroblasts | PTN | NCL | 0.0932056421983309 | 0 | PTN_NCL | PTN - NCL | PTN | Secreted Signaling | PMID: 28356350; PMID: 25620911 |

**Table S3.** Fibroblasts are the results of the first 3 cell communications between sender and receiver.
