# Supplementary material for: Neutrophil-Fibroblast Crosstalk Drives Immunofibrosis in Sequelae of Pelvic Inflammatory Disease Through Neutrophil Extracellular Traps
Source: Mediators Inflamm. 2025 Nov 11;2025:3113542. doi: 10.1155/mi/3113542 (PMC12626693; doi:10.1155/mi/3113542)
Supplement: Supporting Information 4 — Table S4: Partially differentially expressed genes in fibroblasts. [file 3113542.f4.docx]

| **Gene** | **p_val** | **avg_log2FC** | **pct.1** | **pct.2** | **p_val_adj** |
| --- | --- | --- | --- | --- | --- |
| MMP10 | 0 | 2.80 | 0.26 | 0.03 | 0 |
| COL12A1 | 0 | 2.86 | 0.32 | 0.13 | 0 |
| MMP3 | 0 | 2.73 | 0.12 | 0.02 | 0 |
| COL8A1 | 0 | 5.59 | 0.10 | 0.01 | 0 |
| MMP1 | 0 | 4.74 | 0.06 | 0.00 | 0 |
| MMP2 | 0 | 1.70 | 0.79 | 0.75 | 0 |
| MMP7 | 1.03E-251 | 2.25 | 0.08 | 0.03 | 3.15E-247 |
| MMP27 | 7.36E-185 | 3.96 | 0.03 | 0.00 | 2.26E-180 |
| COL24A1 | 1.44E-148 | 1.92 | 0.08 | 0.04 | 4.43E-144 |
| COL6A6 | 5.28E-112 | -1.04 | 0.02 | 0.05 | 1.62E-107 |
| COL13A1 | 5.03E-53 | 3.11 | 0.01 | 0.00 | 1.54E-48 |
| COL22A1 | 1.40E-28 | 1.66 | 0.01 | 0.01 | 4.30E-24 |
| MMP23B | 6.98E-23 | 1.04 | 0.07 | 0.05 | 2.14E-18 |

**Table S4.** Partially differentially expressed genes in fibroblasts.
